# Does deformation of immobilization devices impact treatment accuracy in thoracic cancer radiotherapy?

**DOI:** 10.1002/acm2.14619

**Published:** 2024-12-18

**Authors:** Lianzi Zhao, Louzheng Zhang, Yiwen Hu, Yang Zhong

**Affiliations:** ^1^ Department of Radiation Oncology Fudan University Shanghai Cancer Center Shanghai China; ^2^ Department of Oncology, Shanghai Medical College Fudan University Shanghai China; ^3^ Shanghai Clinical Research Center for Radiation Oncology Shanghai China; ^4^ Shanghai Key Laboratory of Radiation Oncology Shanghai China

**Keywords:** CT‐linac, set‐up error, thermoplastic mask, thoracic cancer, vacuum cushion

## Abstract

**Background:**

Immobilization devices are essential for maintaining accurate and repeatable patient positioning in radiotherapy. This study aimed to evaluate the setup errors and dosimetric deviation induced by the deformation of immobilization devices in thoracic cancer radiotherapy using CT‐linac.

**Materials and methods:**

A retrospective analysis was conducted on 40 thoracic cancer patients who underwent radiotherapy, using vacuum cushion (VC) and thermoplastic mask (TM) for immobilization. A total of 206 weekly Fan‐beam CT (FBCT) images (4–7 per patient) were analyzed to manually delineate immobilization devices and assess their geometric deformations against setup errors. Dosimetric deviations between the clinical plan (CT‐plan) and the delivered plan (FBCT‐plan) were compared for planning target volume (PTV) and organs at risk (OARs). Correlations between dose variations and setup errors were analyzed in lateral (LAT), longitudinal (LNG), and vertical (VRT) axes.

**Results:**

The conformity of the VC (N_up_) and TM (N_down_) with the patient in simulation CT exhibited moderate to strong correlations with VRT setup errors (N_down_: *r* = −0.484, *p* < 0.01;N_up_: *r* = −0.697, *p* < 0.01). However, intra‐fraction deformation of immobilization devices (in FBCT) showed no significant correlation with setup errors. In the dosimetric analysis of OARs, lung dose parameters (D_mean_, V_5_, V_20_) and heart D_mean_ exhibited a consistent absolute difference with increasing setup errors. Dose variation decreased significantly when errors exceeded 5 mm, particularly in the VRT direction for most PTV indices, with the exception of CI and HI. Spinal cord D_max_ variation correlated significantly with setup accuracy along the LNG axis, but not along other axes.

**Conclusion:**

The conformity of immobilization devices in simulation CT exhibits a stronger correlation with setup accuracy than the deformation of these devices in intra‐fraction FBCT. FBCT is recommended for improving treatment precision through dosimetric assessment and planning adjustments.

## 1 INTRODUCTION

Thoracic tumor, including lung and esophageal cancers, are among the most prevalent and frequently diagnosed malignancies in China.^1^, ^2^ Radiotherapy has emerged as a critical and effective treatment modality for these cancers. The precision of therapy is significantly challenged by physiological factors such as respiratory motion, cardiac contractions, large vessel pulsations, and diaphragmatic activity.^3^ Additionally, the duration of radiation treatment necessitates repeated patient positioning, underscoring the importance of utilizing robust immobilization devices to ensure the repeatability and stability across treatment fractions.

Setup error, which reflects the discrepancy between the patient's actual position during each fraction and the initial simulation position, is a key factor contributing to variations between the planned and delivered dose to the planning target volume (PTV) and organs at risk (OARs). Setup error consists of both systematic and random components.^4^, ^5^ While systematic errors have been substantially reduced through the implementation of various patient immobilization devices, random errors remain a significant factor influencing the precision of radiotherapy. For patients with thoracic tumors, the use of thermoplastic masks (TM) and vacuum cushions (VC) has proven particularly effective in minimizing random errors, thereby enhancing patient comfort, stability, and positional reproducibility throughout the course of treatment.

Image‐guided radiation therapy (IGRT) plays a pivotal role in further reducing random errors by acquiring images before each fraction, enabling visualization of the region of interesting (ROI).^6^, ^7^ Common IGRT technologies include electronic portal imaging devices (EPID)^8^, ^9^ and cone‐beam computed tomography (CBCT).^10^, ^11^ Compared to EPID, CBCT offers higher image resolution, lower additional radiation dose, and more accurate three‐dimensional spatial localization, making it increasingly prevalent in clinical practice.^12^, ^13^, ^14^ Studies have demonstrated that CBCT not only facilitates the automatic correction of setup errors but also helps identify factors contributing to random errors, including patient age, body mass index (BMI), and technician proficiency.^15^, ^16^, ^17^ However, most existing research has focused on patient characteristics and technician‐related factors, with limited attention to the impact of immobilization device deformation on setup errors.

Moreover, evaluating the congruence between planned and delivered doses using CBCT presents several challenges. One key issue is that accurate dose calculations require quantitative information on tissue electron density, which is difficult to extract from low‐contrast CBCT images. Additionally, CBCT may not accurately capture the deformation of the PTV and OARs compared to planned CT images, potentially leading to deviations in accumulated dose calculations.^18^

This study leverages the high‐resolution imaging capabilities of the FBCT integrated into the United Imaging uRT‐linac 506c accelerator.^19^, ^20^ By delineation TM and VC across different fractions, we aim to investigate the impact of immobilization device deformation and its rate of change on patient setup errors. Using the United Imaging TPS system, we registered and replicated the clinical treatment plan onto FBCT images to compare the delivered doses with those from the clinical plan, thus evaluating the influence of setup errors on dose delivery.

## 2 MATERIALS AND METHODS

### 2.1 Patient data

This retrospective study enrolled a cohort of 40 patients diagnosed with thoracic tumors, all of whom underwent radiation therapy at the Fudan University Shanghai Cancer Center between July 2022 and March 2023. Each patient was immobilized in the supine position using individualized TM and VC. The detailed characteristics of the patients are summarized in Table 1. Ethical approval for this study was obtained from the Ethics Committee of Fudan University Shanghai Cancer Center.

**TABLE 1:** The characteristics for patients (*n* = 40).

Patient information	
Tumor type and stage	
Lung cancer	20 (III A‐IV B)
Esophageal cancer	20 (I B‐IV B)
Field length (cm)	
Median (range)	14.0(2.9–29.1)
PTV (cc)	
Median(range)	512.1 (14.4–1415.6)
Age (years)	
mean	59.1 ± 11.4
Range	34～78
Gender	
Male	31
Female	9
BMI (body mass index, $\mathrm{kg}/m^{2}$)	
MBI < 18.5	2
18.5 ≤ BMI < 24	25
24 ≤ BMI < 28	9
BMI ≥28	4

### 2.2 Simulation and treatment planning

**FIGURE 1:**
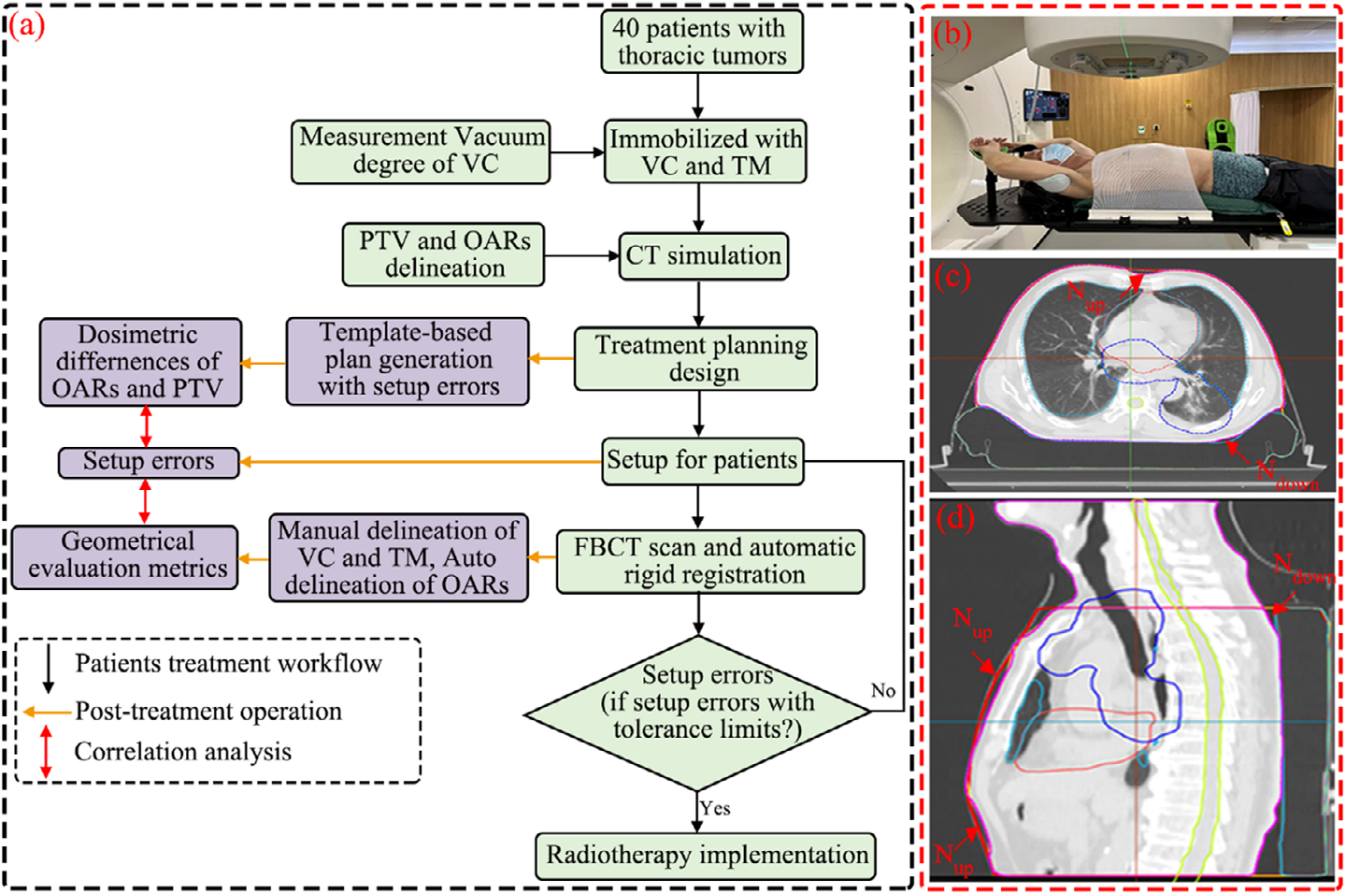
(a) Workflow of patient treatment and post‐treatment procedures in this study; (b) Example of immobilization and localization systems utilizing the VC and TM; (c) and (d) Illustrations showing the gap between the immobilization devices and the patient's body. The contours of the PTV, heart, and lungs are depicted by the blue, pink, and cyan lines, respectively.

The experimental design process is depicted in Figure 1a. For the simulation, all patients were instructed to lie flat in a supine position on a multifunctional board (Klarity Medical & Equipment Co. Ltd., Guangzhou, China) with low‐density headrests. The board was adjustable to ensure comfortable positioning of the armrest and wrist. Each patient was then immobilized using an individualized TM and VC (WFR Aquaplast Corp., Wickoff, New Jersey, USA) as shown in Figure 1b. During the fabrication process, the VC was stabilized by extracting air to a preset pressure of ‐0.075 kPa. In subsequent treatment fractions, the integrity of the VC was assessed weekly, and its vacuum level was monitored to maintain its integrity and stability.

The simulation was performed using a Philips Brilliance Big Bore CT simulator (Philips Healthcare, Amsterdam, Netherlands), with a scanning range extending from 2 cm above the jaw to the lower edge of the liver. Scanning parameters included a slice thickness of 3 mm, a field of view of 45 × 45 cm, a voltage of 120 kV, and an x‐ray tube current of 250 mA. The acquired images were uploaded to the United Imaging Treatment Planning System (UIH) (United Imaging Healthcare, Shanghai, China) for radiotherapy treatment plan development. The ROI were delineated by two or more senior physicians experienced in thoracic cancer. The final radiotherapy plan was reviewed and approved by physicians, as outlined in Table 2.

**TABLE 2:** The ROI constraint functions and dosimetric evaluation metrics.

ROI	Prescription	Constraints or objectives	Dosimetric evaluation
PTV	50.40 Gy/28F, 60 Gy/30F 55 Gy/22F 66 Gy/30F	D_95 _> Prescription, D_2 _< 110% Prescription, Uniform dose = Prescription	D_mean_, D_max_, D_min,_ D_5_, D_95_, CI, HI
Heart	/	Mean Dose < 30 Gy	D_mean_
Lungs‐PTV	/	$D_{\mathrm{mean}}<15$ Gy,V_5_ < 65%, V_20_ < 30%	V_5_, V_20_, D_mean_
SpinalCord	/	D_max_ < 45 Gy	D_max_

### 2.3 FBCT images acquisition and immobilization devices contouring

Prior to each treatment fraction, orthogonal kilovoltage x‐ray imaging or low‐dose FBCT was performed for position verification. FBCT images were acquired once weekly, with scanning parameters including a voltage of 120 kV and a current of 50 mAs. The scanning range was consistent with that of the simulation CT. These FBCT images were rigidly registered with the initial planned CT images to assess setup errors in the LAT, LNG, and VRT principal axes. If the setup error fell within the clinically acceptable threshold, treatment proceeded with bed adjustments. In cases where the error exceeded this threshold, manual repositioning of the patient and an FBCT rescan were required. Additionally, patients were monitored daily for significant weight loss (≥20%), which, if observed, necessitated replanning. To investigate the relationship between the geometric deformation of immobilization devices and setup errors during the course of radiotherapy, the VC and TM were delineated on the FBCT images.

### 2.4 Geometric metrics for immobilization devices

To assess the impact of immobilization devices deformation on setup errors in thoracic cancer patients, we defined the conformity between body and VC (N_down_) and TM (N_up_) using the following formulas:

(1) $$N_{\mathrm{up}}\;=1\frac{V_{\mathrm{up}}}{V_{B}}$$

(2) $$N_{\mathrm{down}}=1\frac{V_{\mathrm{down}}}{V_{B}}$$

where V_B_ represents the volume of the body, V_up_ denotes the volume between TM and body, and V_down_ is the volume between the body and the VC. The degree of conformity (N) ranges from 0 to 1, with values closer to 1 indicating better conformity. An N value of 1 signifies that there is no gap between the patient's body and the VC or TM.

To analyze the variation in geometric metrics of the VC during the course of radiation treatment, we manually delineated the VC on both the simulation CT and the 206 FBCT images form 40 patients, with FBCT scans perform once weekly (4–7 images per patient). Rigid registration of the FBCT and simulation CT was performed, and the VC was copied to the simulation CT. Subsequently, the simulation CT dataset was imported into MIM software (MIM Software Inc., USA) for the computation of the following metrics: Dice similarity coefficient (DSC), Jaccard similarity coefficient (JAC), Hausdorff distance (HD), and mean distance to agreement (MDA). DSC and JAC quantify the overlap between contours A and B, while HD and MDA measure the maximum and mean 3D distances between these contours, respectively. These metrics are defined as follows:

(3) $$\mathrm{DSC}\;=2*\frac{\left|A\cap B\right|}{\left|\left.A\right|\right.+\left|\left.B\right|\right.}$$

(4) $$\mathrm{JAC}\;=\frac{\left|A\cap B\right|}{\left|\left.A\cup B\right|\right.}$$

The DSC and JAC values range from 0, indicating no spatial overlap between the two segmentations, to 1, indicating complete overlap.

(5) $$\mathrm{HD}\;\left(A,B\right)=\max\left\{H\left(A,B\right),H\left(B,A\right)\right\}$$

(6) $$\mathrm{MDA}\;\left(A,B\right)=\frac{H{\left(A,B\right)}_{mean}+H{\left(B,A\right)}_{mean}}{2}$$

(7)

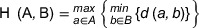



(8)

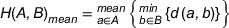

Where d(a,b) represents the 3D Hausdorff distance between point a from contour A and point b from contour B.

### 2.5 FBCT plan design and dosimetric indices difference evaluation

We redesigned the treatment plan using FBCT images, adhering to the original plan template and maintaining identical planning parameters and optimization conditions as in the clinical plan. The delineation of OARs was performed using the intelligent contouring module provided by UIH,^21^ while the PTV was transferred from the simulation CT via image registration. To assess the impact of setup errors on dose distribution, dose calculations were conducted without correcting for setup errors in the VRT, LNG, and LAT directions. For each direction, the absolute values of patient setup errors were categorized into six groups (The distribution in the other two directions is what we consider to be Gaussian.):

Group A: 0 ≤ |x| ≤ 1 mm,
Group B: 1 mm < |x| ≤ 2 mm,
Group C: 2 mm < |x| ≤ 3 mm,
Group D: 3 mm < |x| ≤ 4 mm,
Group E: 4 mm < |x| ≤ 5 mm,
Group F: |x| > 5 mm.

Dose analysis was performed to evaluate the dosimetric differences between the FBCT‐based plan and the clinical treatment plan. For OARs, the maximum dose was used for serial organs, while the mean dose or volume of radiation received was evaluated for parallel organs. For the PTV, dosimetric evaluation metrics included mean dose (D_mean_), maximum dose (D_max_), minimum dose (D_min_), homogeneity index (HI),^22^ and conformity index (CI).^23^ All dosimetric indices are listed in Table 2. The CI and HI were calculated using the following formulas:

(9) $$\mathrm{HI}\;=\frac{D_{5}}{D_{95}}\;$$

(10) $$\mathrm{CI}\;=\frac{V_{R}*V_{R}}{V_{T}*V_{dose}}$$

where D_X_ refers to the dose delivered to X% of the target volume. V_T_ represents the total volume of the target, V_dose_ is the volume enclosed by the reference isodose line, and V_R_ is the volume of the target that is covered by the reference isodose line.

### 2.6 Statistical analysis

Statistical analyses were conducted using IBM SPSS Statistics 23.0 (IBM SPSS Inc., Chicago, Illinois, USA). Two‐tailed Spearman correlation tests (with a 95% confidence interval) were employed to evaluate correlations between variables. Paired *t*‐tests and Wilcoxon signed‐rank tests were used to compare reference and alternative groups, with *p* values less than 0.05 considered statistically significant. For data that followed a normal distribution, comparisons between two independent samples were performed, while nonparametric Mann–Whitney *U* tests were applied for non‐normally distributed data.

## 3 RESULTS

### 3.1 Vacuum degree of VC over time

**FIGURE 2:**
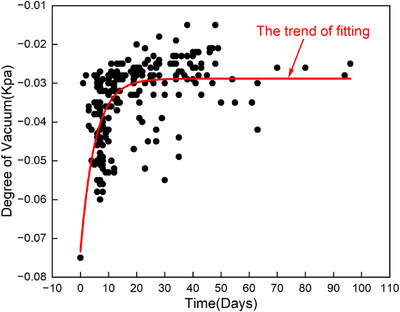
The variation in the vacuum degree of the VC over time.

The vacuum degree of the VC was monitored weekly for all 40 patients. The results demonstrated a gradual decline in the vacuum degree following initial fabrication. However, after approximately 2 weeks, the vacuum levels stabilized within a consistent range, and remained steady for the duration of the treatment course, as illustrated in Figure 2.

### 3.2 The impact of immobilization devices' conformity in simulation CT on mean setup errors

**FIGURE 3:**
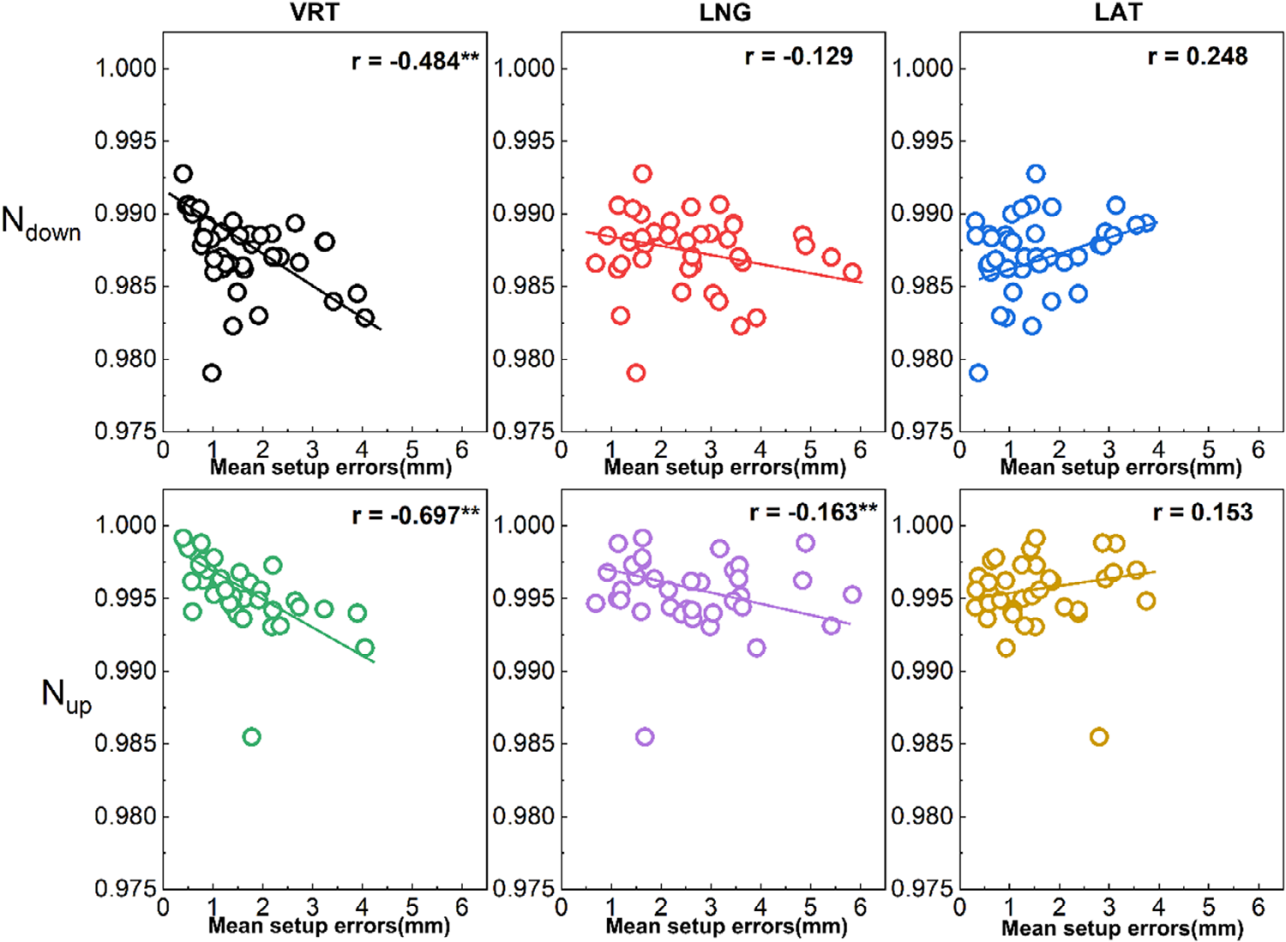
Correlation analysis between mean value of absolute setup error for each patient and immobilization devices conformity in simulation CT (*r*, *n* = 40). ^**^ *p* < 0.01.

The mean VRT setup error values for patients demonstrated a strong negative correlation with the conformity index N_down_ (*r* = ‐0.484, *p* < 0.01) and a moderate negative correlation with the conformity index N_up_ in the simulation CT (*r* = ‐0.697, *p* < 0.01). Conversely, the LAT and LNG mean setup error values showed weaker or no correlation with the N_down_ and N_up_ values, as depicted in Figure 3.

### 3.3 Correlation analysis between geometric indices of immobilization devices and setup errors within intra‐fraction

**FIGURE 4:**
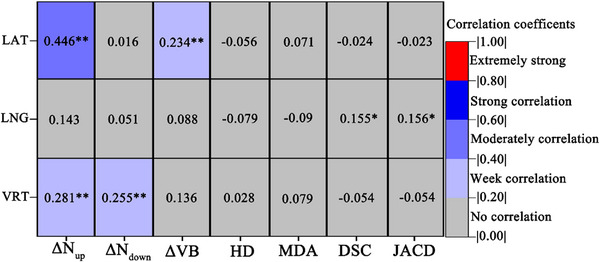
Correlation analysis between setup errors and the rate of change in geometric parameters of the VC in FBCT (*r*, *n* = 280). ΔN_up/down_ (ΔN_up/down _=$\;|\frac{N_{0}-N_{1∼7}}{N_{0}}|\times 100\%$) represents the conformity change rate, where N_x_ is the conformity of the VC in the x‐th fraction. ΔVB (ΔVB =$\;|\frac{VB_{0}-VB_{1∼7}}{VB_{0}}|\times 100\%$) denotes the volume change rate, where VB_x_ is the volume of the VC in the x‐th fraction. ^**^ *p* < 0.01.

Figure 4 illustrates the correlation between changes in geometric parameters of the VC and setup errors during fractionated treatment. The LAT setup error showed a moderate positive correlation with the ΔN_up_ value (*r* = 0.407, *p* < 0.05). However, setup errors in other directions, as well as changes in conformity, volume, and other geometric parameters of the VC, demonstrated weak or no correlation.

### 3.4 Evaluation of dosimetric impact of setup errors

**FIGURE 5:**
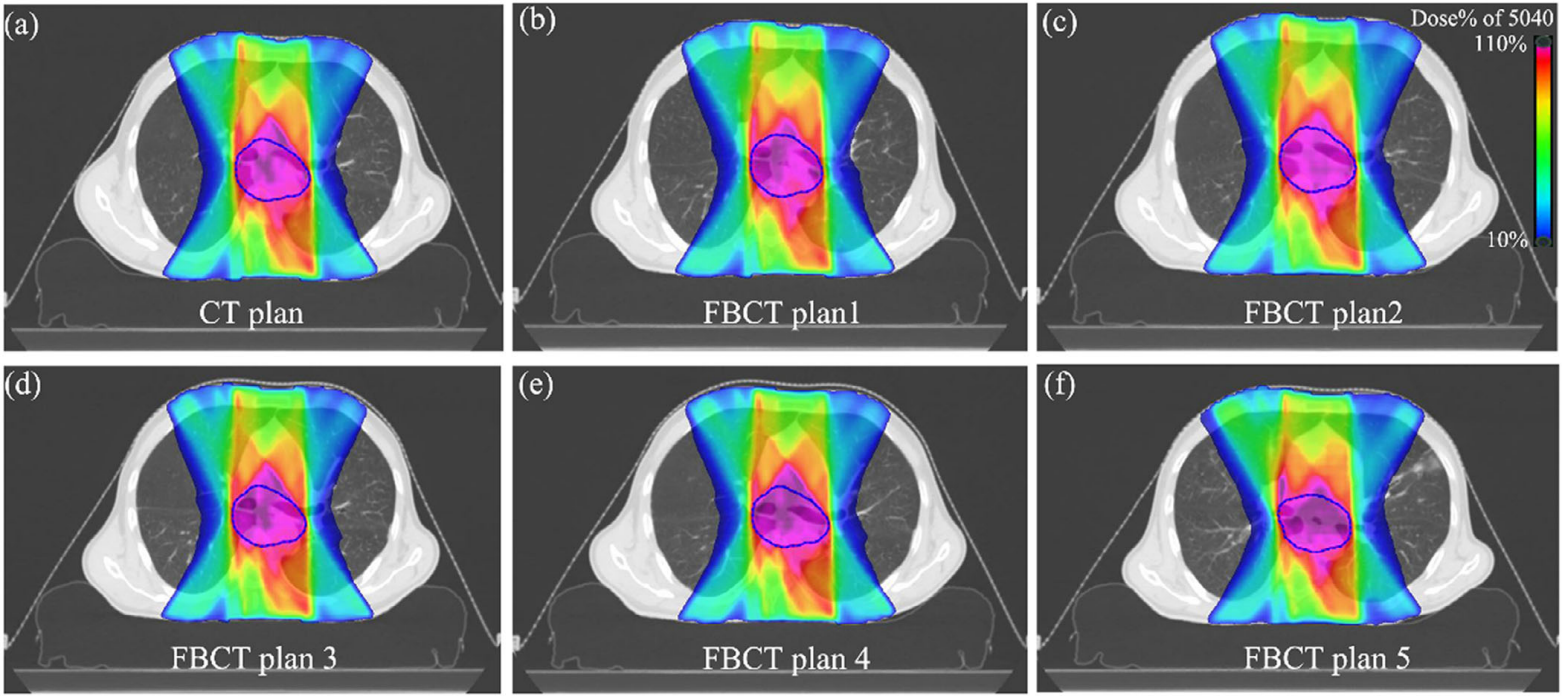
Dose distribution in a cross‐sectional view for a lung cancer patient (prescription: 5040 cGy) on (a) CT image and recalculated dose distribution with setup errors on FBCT; (b)‐(f) for different fractions.

**FIGURE 6:**
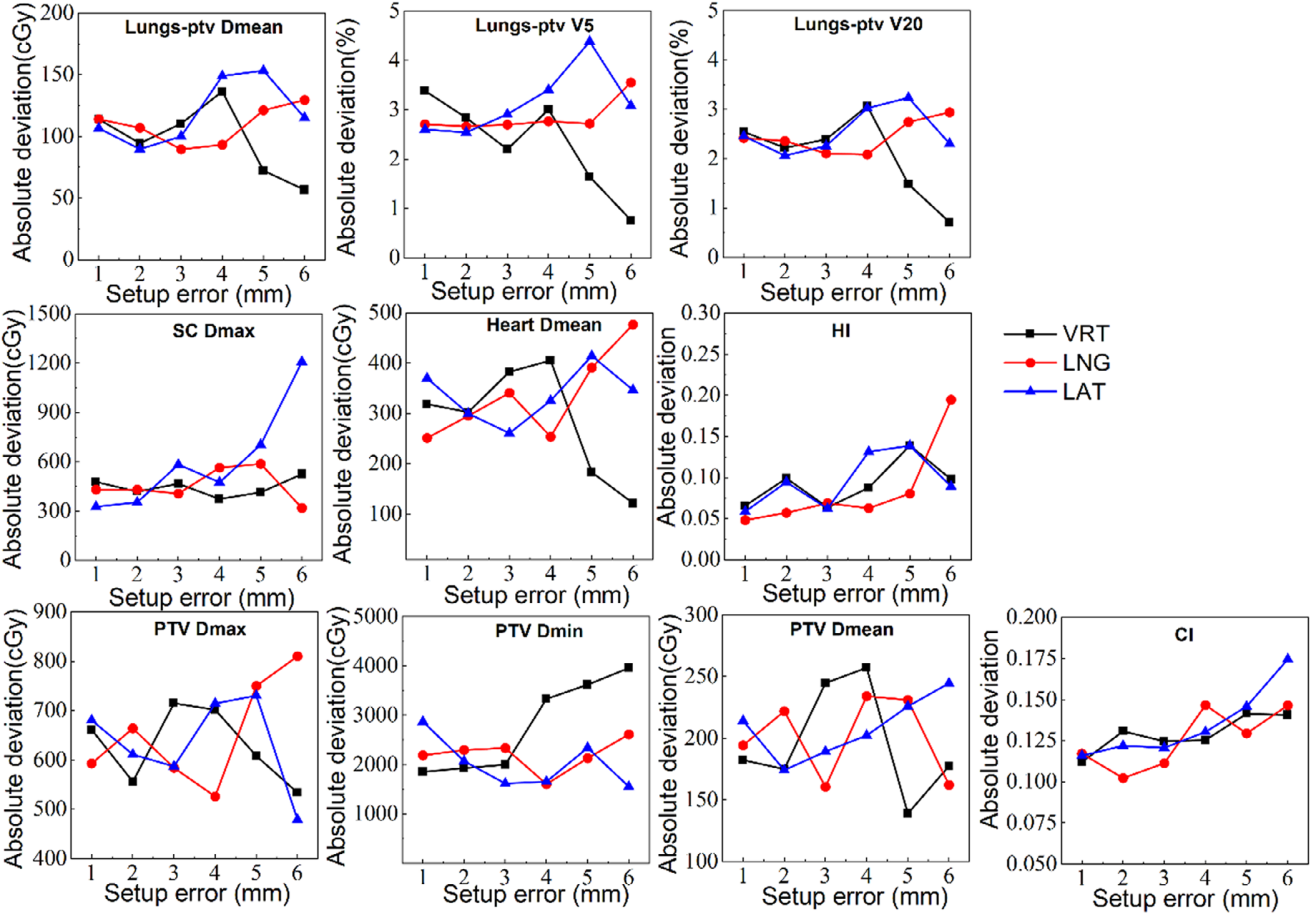
The trend of dosimetric indices change rate in PTV and OARs with setup errors in three directions. The dose change is defined as the absolute value of the difference between the dose parameters of each FBCT plan and those of the planning CT plan.

Recalculated dosimetric parameters were compared to reference values, as demonstrated in a representative case in Figure 5. The correlation between the dose variation and setup errors is illustrated in Figure 6. For normal tissues, particularly the lungs, dosimetric parameters such as Lungs‐PTV D_mean_, Lungs‐PTV V_5_, and V_20_ exhibited a consistent absolute difference in dose variation within the tolerance of 5 mm setup errors. Notably, dose variation decreased significantly when errors exceeded 5 mm in the LAT and VRT directions. This phenomenon was also evident in the D_mean_ of the heart. The dose variation rate for SC D_max_ demonstrated a significant positive correlation with setup errors in the LAT direction, while dose variation in the VRT and LAT directions exhibited fluctuating trends with changes in the magnitude of errors. For the PTV dosimetric parameters, CI and HI demonstrated increased variability with increasing setup errors in all three directions. However, no significant correlations were observed for dose deviations in D_max_, D_min_, and D_mean_. Notably, significant changes in dose deviation were also observed when setup errors exceeded a threshold of 5 mm in any direction.

## 4 DISCUSSION

In this study, we utilized the high‐quality imaging capabilities of FBCT integrated into the United Imaging uRT‐linac 506c accelerator to evaluate the impact of immobilization device deformation on setup errors during thoracic cancer radiation therapy. Our analysis revealed that the conformity of the immobilization devices during CT simulation significantly influences setup accuracy in the VRT direction, but shows no significant correlation with setup errors in the LAT and LNG directions. Additionally, inter‐fractional deformation of the immobilization devices showed minimal impact on the setup errors. Notably, for the relationship between setup errors and dose variations, we identified that increased setup errors significantly impact most dosimetric evaluation metrics, particularly when setup errors exceed 5 mm.

Immobilization devices plays a critical role in ensuring accurate and repeatable patient positioning throughout radiotherapy. The use of VC and TM for immobilization is widespread, given their ability to enhance patient comfort and positioning consistency, particularly in stereotactic body radiation therapy (SBRT).^24^, ^25^ To mitigate deformation risks, we monitored the vacuum levels of the VC throughout the treatment course, finding no significant deformation due to air leakage (Figure 2). This consistent performance highlights the importance of routine vacuum checks to prevent setup inaccuracies caused by cushion deformation.

Advances in radiotherapy equipment have significantly reduced systematic errors, placing greater emphasis on managing random errors during treatment. IGRT is an essential tool in mitigating random setup errors, with CBCT being a prevalent imaging modality.^26^, ^27^, ^28^ However, CBCT has limitations in dose accuracy and adds unnecessary radiation exposure when frequently employed. The uRT‐linac 506c system, with its integrated FBCT, offers distinct advantages over CBCT, including superior image quality, faster scanning times, and improved soft tissue visualization. This integration enables real‐time dose recalculation based on treatment images, overcoming challenges associated with electron density discrepancies in CBCT‐based calculations.

In this study, we took advantage of the FBCT system's capabilities to re‐register and transfer the clinical treatment plan onto the FBCT images, recalculating the dose distribution without correcting for setup errors. By assessing the actual dose delivered, we identified significant correlations between setup errors and dose variation for both OARs and the PTV. This approach allowed for a more accurate analysis of the dosimetric impact of setup errors, particularly those exceeding 5 mm, which were associated with considerable deviations from the planned dose.

While deformation of the VC and TM was hypothesized to influence setup accuracy, our results showed limited correlation between VC deformation and setup errors during the course of treatment. This lack of significant correlation may be attributed to the careful maintenance of vacuum levels throughout the treatment process, as shown in Figure 2. Despite this, we found that variations in body volume (VB), influenced by changes in patient weight, impacted the conformity between the body and TM, particularly the upper portion (N_up_). As demonstrated in Table 3, changes in VB significantly correlated with N_up_ and its rate of change (ΔN_up_), reinforcing findings from previous research on weight fluctuations and their effect on setup accuracy.^29^

**TABLE 3:** Correlation analysis between VC conformity change rate and body volume change rate, VC geometric parameters (*r*, *n* = 280).

	N_up_	ΔN_up_	N_down_	ΔN_down_
ΔV_B_	−0.479^**^	0.466^**^	−0.050	0.197^**^
HD (mm)	0.025	0.006	0.062	−0.001
MDA (mm)	0.044	0.013	−0.219^**^	0.065
DSC	−0.118	0.027	0.262^**^	−0.056
JACD	−0.119	0.028	0.262^**^	−0.054

** *p* < 0.01.

Several limitations of our study should be noted. First, setup errors were only analyzed in three translational dimensions (VRT, LNG, LAT); rotational errors (pitch, roll, and yaw) were not investigated. Second, ORI delineation on FBCT was performed using an automated contouring module, which may have introduced variability in inter‐fractional organ contours. Additionally, the target area contouring on FBCT was achieved through rigid registration with the simulation CT, rather than manual re‐contouring on each FBCT by an oncologist. This may have affected the accuracy of the dose analysis for the target area, representing a limitation in the assessment of dose deviations in this study.

## 5 CONCLUSION

In this study, we employed the diagnostic‐quality FBCT imaging capabilities of a CT‐integrated linear accelerator to evaluate the influence of geometric changes in immobilization devices on setup errors during radiotherapy for thoracic tumors. Additionally, by redesign the clinical treatment plan parameters onto registered FBCT images, we recalculated the delivered dose and analyzed the correlation between setup errors and variations in dosimetric parameters for the PTV and OARs. This methodology provides a more accurate assessment of discrepancies between the planned and delivered doses in fractionated radiotherapy, accounting for setup errors and ROI deformation. The approach facilitates a more precise evaluation of treatment outcomes and offers valuable insights for subsequent treatment planning, thereby improving the overall effectiveness and accuracy of radiotherapy.

## AUTHOR CONTRIBUTIONS

Study concept and design (Lianzi Zhao, Louzheng Zhang, Yang Zhong); acquisition of data (Lianzi Zhao, Yiwen Hu); analysis and interpretation of data (Lianzi Zhao, Louzheng Zhang, Yang Zhong); drafting of the manuscript (Lianzi Zhao, Louzheng Zhang, Yang Zhong). All authors read and approved the final manuscript.

## ETHICS STATEMENT

This study was approved by the Fudan University Shanghai Cancer Center Institutional Review Board, and all methods were performed in accordance with the guidelines and regulations of this ethics board. Informed consent was obtained from all individual participants included in the study.

## CONFLICT OF INTEREST STATEMENT

The authors declare no conflicts of interest to disclose.

## Data Availability

The data that support the findings of this study are available from the corresponding author upon reasonable request.
